# Comparative Approaches in Treating Double-J Stent Syndrome: Monotherapy or Combination Therapy?

**DOI:** 10.3390/jcm13144278

**Published:** 2024-07-22

**Authors:** Cătălin Pricop, Carina Alexandra Bandac, Marius Ivanuță, Daniel Rădăvoi, Viorel Jinga, Dragoş Puia

**Affiliations:** 1 “Grigore T Popa” University of Medicine and Pharmacy, 700115 Iasi, Romania; 2 Department of Urology, “Dr. C.I. Parhon” Clinical Hospital, 700503 Iasi, Romania; 3 Department of Urology, “Theodor Burghele” Clinical Hospital, 050653 Bucharest, Romania; 4 “Carol Davila” University of Medicine and Pharmacy, 050653 Bucharest, Romania

**Keywords:** ureteral stent-related symptoms, tamsulosin, solifenacin, mirabegron, desloratadine

## Abstract

**Introduction**: The application of double-J ureteral stents in urology is widespread, but their use is often accompanied by complications and bothersome symptoms, affecting patients’ quality of life (QoL). While various medications have been tested for alleviating the symptoms associated with double-J stents, consensus on their effectiveness remains elusive. This study aims to investigate the effectiveness of tamsulosin, solifenacin, mirabegron, desloratadine, and combination therapy using a Romanian-adapted version of the Ureteral Stent Symptom Questionnaire (USSQ). **Materials and Methods**: A prospective, observational, randomised trial was conducted at the Urology and Renal Transplant Clinic of Dr. “C.I. Parhon” Clinical Hospital in Iasi between 1 January 2022 and 1 August 2023. Three hundred twenty seven patients who underwent their first double-J stent insertion were evaluated with the Romanian-adapted USSQ at baseline and 30 days post-insertion. Patients were randomly divided into six groups based on the prescribed medications: control, tamsulosin, mirabegron, solifenacin, desloratadine, and combination therapy. **Results**: The data suggest a significant reduction in symptoms in patients who received medication compared with the control group. Furthermore, the combined medication of solifenacin 10 mg and tamsulosin 0.4 mg was particularly effective in reducing pain with statistical significance compared to the control group (*p* = 0.001). The highest mean scores for urinary symptom severity were observed in the control group (12.37 ± 6.82), and the lowest was in the mirabegron group (9.94 ± 5.82). The individuals who received a daily dose of 50 mg of mirabegron saw the most notable influence on their job. **Conclusions**: While no single medication emerged as a “miracle drug” for managing symptoms related to double-J stent insertion, the combination therapy of solifenacin and tamsulosin is the most promising option for improving symptoms related to double-J stent insertion and QoL. Additional extensive research is required to validate these initial results.

## 1. Introduction

A double-J (JJ) stent is a radiopaque catheter of varying lengths and thicknesses made from different polymeric materials and placed endoscopically to maintain ureteral patency, thereby facilitating urine drainage [1,2]. Ureteral catheterisation and JJ stent insertion have become fundamental components in modern urology, with many indications [3].

Despite its many advantages, the prolonged use of JJ ureteral stents is often associated with complications and, in a significant percentage, troublesome symptoms for patients [4]. The most common complications include mechanical issues such as stent fracture, stent migration, or calcification and discomfort due to irritation of the bladder mucosa, particularly the trigone, by the distal loop of the stent or due to smooth muscle spasms or ureteral reflux [5,6,7,8].

Unfortunately, the complications and discomfort associated with JJ stents affect a significant number of patientsaccording to Joshi et al., as high as 80% [4]. Conversely, other studies, such as the one by Hao et al., have reported a much lower incidence of complications (19.6%) [9].

JJ stent syndrome refers to symptoms experienced by some patients with these stents. The symptoms can vary in intensity and duration and most commonly include lower urinary tract symptoms (LUTS) such as urinary frequency, urgency, nocturia, and dysuria. Other common symptoms among these patients include bladder emptying pain, flank pain, macroscopic haematuria, urinary incontinence, and sexual dysfunction [10,11].

Existing literature suggests that some medications already used in urology for various conditions may alleviate the discomfort of patients with JJ stents. The drug therapies studied so far are alpha-blockers, antimuscarinics, beta-3 adrenergic agonists, and antihistamines [12,13,14].

Based on patient history and the Romanian-adapted version of the Ureteral Stent Symptom Questionnaire (USSQ), our study aims to identify the primary issues faced by patients who have undergone double-J stent insertion. Although many studies have evaluated various drugs or combinations for the relief of stent syndrome, they have not reached definitive results. Therefore, the best treatment option is still debatable. Most studies suggest the use of alpha-blockers and anticholinergic medications as effective treatments for stent-related symptoms. At the same time, the European Association of Urology (EAU) guidelines recommend a combination of pharmacological and non-pharmacological approaches to manage stent-related discomfort effectively. In addition to alpha-blockers and anticholinergics, we aimed to evaluate desloratadine, mirabegron and combinations of drugs. We also assessed the occurrence of Lower Urinary Tract Symptoms (LUTS) and the efficacy of tamsulosin, solifenacin, mirabegron, desloratadine, and combination therapy with solifenacin and tamsulosin in controlling the arising symptomatology and improving the QoL.

## 2. Materials and Methods

The present study is a prospective, interventional, randomised trial conducted from 1 January 2022 to 1 August 2023 at the Urology and Renal Transplant Clinic of Dr. “C.I. Parhon” Clinical Hospital in Iasi. The study received approval from the Hospital Ethics Committee and was registered under number 3339/2021.

Unique Inclusion Criteria: We considered patients who were undergoing their first JJ stent insertion, regardless of the stent’s dimensions, who completed our Romanian-adapted version of the USSQ questionnaire, and who consented to inclusion in the study.

Exclusion Criteria: Non-compliant patients, those who declined participation, patients with various forms of dementia/mental retardation, pregnant women, patients with uncorrected coagulation disorders, those with tumour pathology, patients known to have overactive/neurological bladder, men suffering from benign prostatic hyperplasia, patients in severe general condition upon presentation, those with recurrent UTIs or previous MDR UTIs, and those who already were taken alpha-blockers, antimuscarinics, mirabegron, or desloratadine were excluded.

The length of the JJ stent was chosen according to the patient’s height, so for those shorter than 160 cm, a 22 cm stent was used; for those between 161 and 175 cm, a 24 or 26 cm; and for those over 175 cm, a 26 cm stent. Patients were asked to complete an informed consent form indicating their agreement to participate in the study and acknowledging their understanding of its procedures, potential medications, side effects, and the randomised allocation to study groups based on the treating physician’s decision. Additionally, patients received an informational brochure about the ureteral stent.

Patients were evaluated using our Romanian-adapted version of the USSQ questionnaire at JJ stent insertion and on day 30 post-insertion and VAS for pain.

Patients were randomly divided into six study groups, and medication for each patient was prescribed by the attending urologist: the control group—patients who were not recommended any medication for symptom control; the tamsulosin group—patients recommended tamsulosin 0.4 mg/day for 30 days; the mirabegron group—patients recommended mirabegron 50 mg/day for 30 days; the solifenacin group—patients recommended solifenacin 10 mg/day for 30 days; the desloratadine group—patients recommended desloratadine 5 mg/day for 30 days; and the combination therapy group—patients prescribed tamsulosin 0.4 mg/day and solifenacin 10 mg/day for 30 days. Patients were assigned to one of the six treatment groups using computer-generated random numbers to ensure an unbiased allocation.

All data underwent statistical analysis to establish correlations between various classes of medications and observed symptoms. Descriptive statistics were employed, with categorical variables expressed as frequencies and percentages, while numerical variables were described using means and standard deviations. Bar graphs were also utilised to offer a visual representation of the data distribution.

In the comparative analysis of the six treatment groups for patients with JJ stent insertion, the Analysis of Variance (ANOVA) test was utilised. The significance level was determined using the associated probability (*p*-value), with a value below the predetermined significance level indicating that the test was statistically significant.

Statistical analyses were conducted using the SPSS software for Windows, version 2.20 (IBM Corp., Armonk, NY, USA).

All experimental procedures and protocols used were in accordance with Directive 210/63/EU. The Ethics Committee of Dr. C.I. Parhon Clinical Hospital granted ethical approval for the study. Written consent was secured from all participating patients.

## 3. Results and Discussions

**Figure 1:**
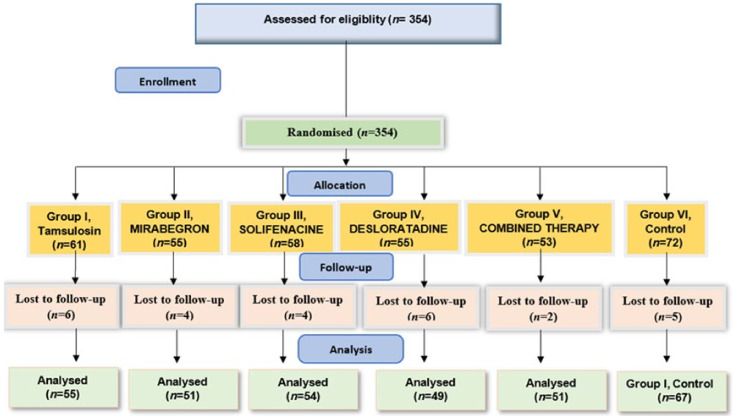
Distribution of patients across the groups.

Initially, 354 patients were enrolled in the study, distributed across the six groups. Of these, 327 completed our study. The distribution across the groups is shown in Figure 1.

Regarding patient gender distribution across the six groups, females were the majority in five of the groups. The exception was the group treated with tamsulosin 0.4 mg per day, in which males constituted 61.8%, and females made up 38.2% of the total participants, even if the enrolment was randomised.

Regarding the place of origin, a significant proportion of patients from urban environments were observed in groups that received no treatment and those treated with mirabegron or desloratadine. Conversely, 51% of patients who were administered tamsulosin originated from rural areas. This rural demographic was least represented among groups receiving combination therapy and solifenacin. In the group where no medication was prescribed for symptom control, most participants were women, comprising precisely *n* = 41 of the group. The fewest number of men were observed in the solifenacin group (Table 1).

Regarding age distribution, the control group primarily consisted of patients in the age ranges of 40–59 and 60–79 years, each representing 40.3% of the group’s total population. In the group receiving tamsulosin, the predominant age bracket was 60–79 years, accounting for 43.6% of the group. In the groups treated with mirabegron, solifenacin, and desloratadine, the most common age category was 40–59 years.

In our study groups, no statistically significant difference was observed in terms of age, with a *p*-value of 0.26 between groups.

By analysing the urinary symptoms score, the highest mean value was observed in the control group, registering at 12.37 ± 6.82. The lowest mean value was found in the group treated with mirabegron, at 9.94 ± 5.82. The analysis indicates no statistically significant difference in the treatment effect on urinary symptoms between the control group and the mirabegron, solifenacin, and desloratadine treatment groups. A statistically significant difference was observed between the group receiving combined therapy (solifenacin + tamsulosin) and the control group, with a *p*-value of 0.009.

Concerning pain associated with JJ stent syndrome, the lowest average value was recorded in patients undergoing combined therapy, with a mean value of 2.02 ± 1.33. The highest mean score was observed in patients who received monotherapy with desloratadine, registering at 2.7 ± 1.56. No statistically significant difference was noted in the treatment effect on pain between the control and most treatment groups. The only situation with a statistically significant difference was between the combined therapy group and the control group, with a *p*-value of 0.001. Regarding VAS for pain scores, the highest mean value of 3.37 ± 1.99 was associated with patients prescribed solifenacin. The combined therapy group found the lowest mean value, with a mean of 2.65 ± 1.16. A statistically significant difference in the treatment effect on VAS pain was observed between the combined therapy group and those treated with desloratadine.

Regarding work impact, the most significant impact was observed in patients treated with mirabegron 50 mg daily, with the highest mean value of 4 ± 5.43. This large deviation suggests significant data dispersion or variability. Conversely, solifenacin therapy had the most negligible impact on work quality compared to the other five groups, with a mean value of 2.31 ± 4.71. Furthermore, a statistically significant difference was observed in the impact of treatment on work capacity between the combined therapy group and the mirabegron treatment group, with a *p*-value of 0.035.

The analytical results suggest that there are no statistically significant differences in the treatment effects on overall health, sexual life, or other health-related issues between the control group and the treatment groups receiving tamsulosin, mirabegron, solifenacin, desloratadine, and combination therapy. The only statistically significant difference was observed in the domain of ‘additional problems’ between the combined therapy group and the control group (*p* = 0.001). Furthermore, no significant disparities were observed between these individual treatment options and the group subjected to combined therapy (Table 2).

**Figure 2:**
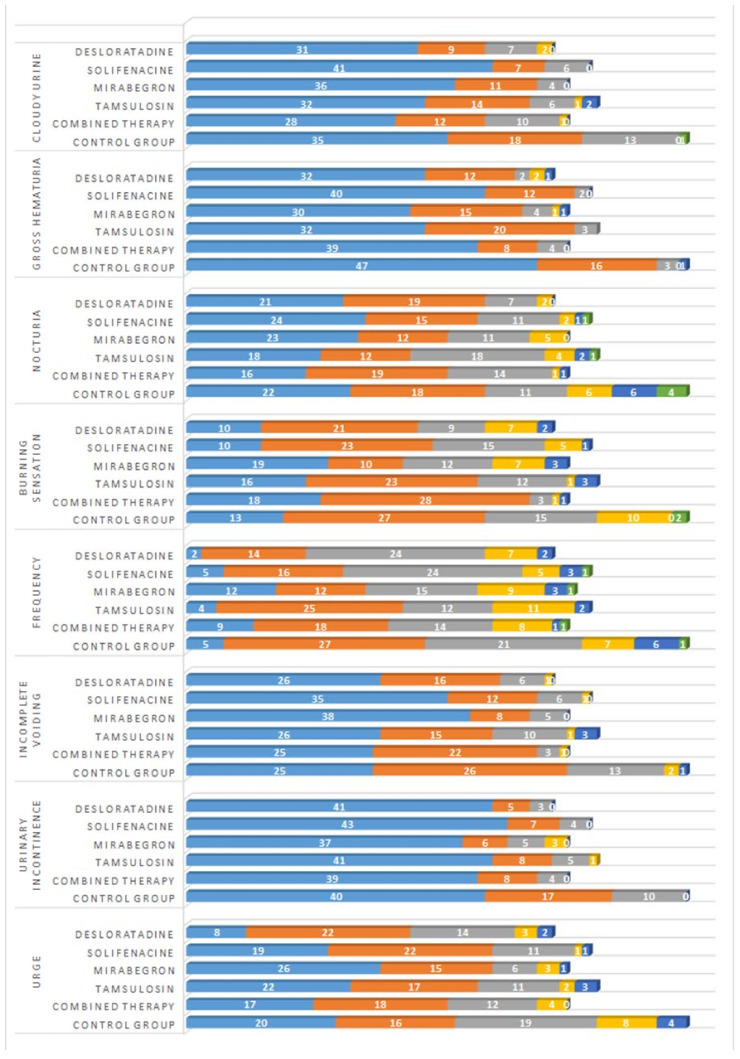
Frequency of urinary symptoms. The number of patients in each study group who reported urinary symptoms as ‘not at all’ (blue), ‘rarely’ (orange), ‘sometimes’ (grey), ‘half of the time’ (yellow), ‘most of the time’ (dark blue), and ‘always’ (green).

Our data suggests that the experiences of urinary system symptoms differ among patients across various treatment groups, as illustrated in Figure 2. Notably, urinary incontinence was uncommon among most patients in all groups; they either did not report this issue or encountered it only rarely. These findings imply that the treatment regimens under study may effectively mitigate urinary incontinence for most patients.

Most patients across all groups reported experiencing polyuria either rarely or occasionally, most notably in the control and desloratadine groups. Similarly, urinary urgency was more frequently reported in these two groups.

Regarding the sensation of burning during urination, most patients did not report severe symptoms irrespective of their treatment group. However, a minority did experience occasional or rare burning sensations. Nocturia (frequent urination during the night) was not a common complaint, although a small subset of patients reported experiencing it either rarely or sometimes. Gross hematuria was also uncommon, except in some isolated instances, primarily within the tamsulosin and mirabegron treatment groups. Lastly, most patients did not report experiencing cloudy urine; however, a small number in the tamsulosin and desloratadine groups indicated experiencing this symptom either rarely or sometimes.

This comprehensive evaluation of urinary symptoms among different patient groups and treatment regimens offers valuable insights into the effectiveness of various pharmacological interventions.

## 4. Discussion

Although it offers numerous advantages, the prolonged use of JJ stents is frequently associated with various complications and, in a significant percentage, bothersome symptoms for patients with indwelling ureteral stents [11,14].

The mechanisms underlying ureteral stent-related symptoms are complex and not completely understood.

The predominant view mainly ascribes the condition to the mechanical disruption caused by the stent, affecting both the ureter and the trigone region of the bladder. This leads to impaired ureteral peristalsis, bladder mucosa inflammation, detrusor muscle spasms, and urinary reflux back into the kidney [15].

The postoperative evaluation and monitoring of patients who have undergone JJ stent insertion is of paramount interest for detecting symptomatology and the degree of discomfort caused by the presence of the stents. A comprehensive assessment that considers multiple parameters, such as diverse symptoms, impact on work, and impact on sexual life, is necessary. Of course, challenges can arise when applying questionnaires to noncompliant patients or those with a limited understanding of their symptomatology and pathology [16].

A series of studies employing questionnaires to evaluate patients with JJ stents have proven useful and highlighted the impact of ureteral catheter insertion on patients. The most well-known questionnaire is the multilingually validated USSQ, introduced by Joshi et al., which comprises questions related to six categories of issues: urinary symptoms, pain, general health status, work performance, sexual life, and other problems [11,17].

A review of the literature data indicates that patients who have had a double-J stent inserted most commonly reported symptoms associated with the urinary tract, such as pain (18–58%), macroscopic haematuria (34–85%), dysuria (13–72%), polyuria (50–85%), and urinary urgency (43–67%). These symptoms can be caused by stent-dependent factors (length, diameter, composition, and shape) or patient-related factors (physical activity and vesicoureteral reflux) [11].

The effective management of stent-related symptoms often involves a combination of pharmacological and non-pharmacological approaches. The most extensively studied treatments have been alpha-blockers and antimuscarinics [18]. However, more research studies are needed in the specialised literature when it comes to the use and efficacy of mirabegron or combination therapy with solifenacin and tamsulosin [3,12,14].

A systematic review of the literature concerning medication used in alleviating discomfort associated with JJ stent placement highlighted the more frequent use of alpha-blockers, followed by solifenacin and mirabegron [19]. Gupta et al. asserted 2010 that botulinum toxin type A significantly reduced stent-related pain and decreased the need for analgesics [13]. Park et al., in 2009, compared tolterodine, alfuzosin, and placebo in a randomised study and indicated that the two medications positively impacted HRQoL [14]. A meta-analysis conducted by Yakoubi et al. in 2011 revealed that four studies demonstrated a significant reduction in JJ stent-induced discomfort using alfuzosin, and seven studies achieved the same results using tamsulosin [20].

A multicentre study conducted in Turkey evaluated the effects of mirabegron on improving the symptoms of patients with a ureteral stent. The study included 145 patients who received a daily dose of 50 mg mirabegron and showed symptom improvement and decreased QoL scores [21].

Antihistamines have also shown potential in alleviating urinary stent-related symptoms. Multiple in-vitro studies have indicated the involvement of H1 receptors in both ureteral peristalsis and bladder contraction [22]. Both first- and second-generation H1 antagonists have shown activity in the urinary tract. First-generation antihistamines are competitive inhibitors of muscarinic receptors and have anticholinergic effects. In clinical practice, antihistamines are commonly used in managing pain caused by interstitial cystitis [23].

Moreover, a randomised controlled study found the efficacy of antihistamines in managing pain in renal colic induced by ureteral stones [24]. New evidence suggests that inflammatory changes in the bladder are associated with eosinophilic reactions in patients with implanted urinary stents. Eosinophilic cystitis is commonly associated with irritative bladder symptoms, indicating a new treatment paradigm for such patients [25]. To date, no published study has evaluated the efficacy of desloratadine in controlling LUTS in patients with a JJ stent.

In a 2019 meta-analysis conducted by Chen et al., three studies involving 238 participants examined the efficacy of tamsulosin monotherapy versus combination therapy with tamsulosin and solifenacin for alleviating stent-related symptoms. The aggregated data revealed no substantial difference between the two treatment approaches regarding urinary symptoms, pain, overall health, work and sexual life, and the total USSQ score compared to our study [26].

In our study, the effectiveness of tamsulosin 0.4 mg, solifenacin 10 mg, mirabegron 50 mg, desloratadine 5 mg, and combination therapy with solifenacin 10 mg and tamsulosin 0.4 mg were investigated using our Romanian-adapted version of the USSQ. The initial characteristics of the patients indicated that females were the predominant gender in five of the six groups. The sole exception was the group administered a daily 0.4 mg dose of tamsulosin, where the majority were males. Regarding age distribution, most patients fell within the 40–59 age range (43.7%), followed by those in the 60–79 age group (40.3%).

Our findings indicate that patients in the groups prescribed medication experienced an improvement in urinary symptoms compared to the control group. However, statistical analysis shows that the differences were not statistically significant in the treatment effect on urinary symptoms between the control group and the mirabegron, solifenacin, and desloratadine treatment groups, nor between the group that received combined therapy (solifenacin + tamsulosin) and the other groups with medication. The difference was statistically significant between the group with combined therapy and the control group (*p* = 0.009). The highest average score was observed in the control group (12.37), while the lowest was in the mirabegron group (9.94). In addition, a study by Lim and colleagues assessed the efficacy of tamsulosin and solifenacin by employing IPSS, VAPS, and QOL surveys. They concluded that monotherapies with either tamsulosin or solifenacin did not demonstrate clear benefits in alleviating most stent-related symptoms. However, the synergistic approach of combining both medications proved substantially more effective [17].

Regarding mean scores for pain domain, the combined therapy group recorded the lowest average score (2.02), which was statistically significant compared to the control group’s (2.6) with a *p*-value of 0.001. No statistically significant differences were observed in the treatment effects on pain between the control group and those treated with tamsulosin, solifenacin, mirabegron, and desloratadine. A statistically significant difference in the effect on VAS pain scores was noted between the group receiving combined therapy and the desloratadine-treated group, with a *p*-value of 0.001.

On the other hand, Palinggi et al. compared the effects of solifenacin with mirabegron and found that solifenacin was more effective in improving the USSQ score [27]. In a prospective randomised study by Yavuz et al., 161 patients were evaluated. The findings showed that the need for analgesics was lower in the tamsulosin (5.1 ± 1.8) and mirabegron (4.5 ± 1.4) groups compared to the control group (5.9 ± 2.1). Additionally, urinary symptoms were less prevalent in the tamsulosin group when compared to the control group [28].

Our study showed no statistically significant differences in overall health or sexual life between the control group and those treated with tamsulosin, mirabegron, solifenacin, desloratadine, or combination therapy.

In the current research, a statistically significant difference in work capacity was observed between the group undergoing combined therapy and the group administered mirabegron, as indicated by a *p*-value of 0.035.

Furthermore, our results indicated that, concerning the sixth domain of the USSQ (additional problems), statistically significant differences were only observed between the combined therapy group and the control group, with a *p*-value of 0.001.

Additionally, a 2017 meta-analysis by Wang et al. encompassed three studies with 197 patients, contrasting the USSQ scores of combination therapy with those of solifenacin monotherapy. The analysis revealed no significant disparities in the overall USSQ score or specific domains such as urinary symptoms, pain, general health, work efficiency, and sexual performance [6].

The limitations of our study stem from several factors: the absence of a separate analysis for patients with bilateral ureteral stent placement, the lack of long-term follow-up data, which is crucial for understanding the enduring efficacy and side effects of the compared treatments, and the omission of considerations regarding the length and thickness of the JJ stent. This is particularly relevant as the existing literature suggests that an overly long JJ stent may predispose patients to symptoms related to ureteral stenting.

## 5. Conclusions

Our study found that a combination therapy of solifenacin 10 mg and tamsulosin 0.4 mg was the most effective in improving urinary symptoms and pain scores, as evidenced by the statistically significant differences from the control group. However, no significant improvements were observed in overall health or sexual life across treatment groups. The combination therapy was also more effective in improving work performance and addressing additional problems than the other groups. A more comprehensive understanding of the efficacy of these treatments for urinary symptoms and other related domains can be achieved through further longitudinal studies, including more extensive and diverse patient populations.

## Figures and Tables

**Table 1:** Demographic characteristics of the patients.

Parameter	Control Group (*n* = 67)	Combined Therapy (*n* = 51)	Tamsulosin (*n* = 55)	Mirabegron (*n* = 51)	Solifenacin (*n* = 54)	Desloratadine (*n* = 49)
	n (%)	n (%)	n (%)	n (%)	n (%)	n (%)
Sex						
Male	26 (38.2%)	23 (45.1%)	34 (61.8%)	19 (37.3%)	15 (27.8%)	18 (36.7%)
Female	41 (60.3%)	28 (54.9%)	21 (38.2%)	32 (62.7%)	39 (72.2%)	31 (63.3%)
Age (years old)						
18–39	11 (16.4%)	5 (9.8%)	10 (18.1%)	12 (23.5%)	12 (22.2%)	6 (11.5%)
40–59	27 (40.3%)	21 (41.1%)	20 (36.3%)	22 (43.1%)	24 (44.4%)	22 (42.3%)
60–79	27 (40.3%)	25 (49%)	24 (43.6%)	16 (31.4%)	18 (33.3%)	20 (38.5%)
80+	2 (3%)	0 (0%)	1 (1.8%)	1 (2%)	0 (0%)	1 (1.9%)
Residential setting						
Rural	32 (47.8%)	26 (51%)	31 (56.4%)	16 (31.4%)	30 (55.6%)	24 (49%)
Urban	35 (52.2%)	25 (49%)	24 (43.6%)	35 (68.6%)	24 (44.4%)	25 (51%)

**Table 2:** Romanian-adapted USSQ mean scores.

	Group	Mean SD	*p*-Value	
			Control Group	Combined Therapy
**Urinary Symptoms**	Control Group	12.37 ± 6.82	-	0.009
	Combined Therapy	10.10 ± 6.27	0.009	-
	Tamsulosin	10.80 ± 6.74	0.36	0.75
	Mirabegron	9.94 ± 5.82	0.57	0.12
	Solifenacin	10.06 ± 5.41	0.68	0.85
	Desloratadine	11.76 ± 6.10	0.67	0.55
**Pain** **Pain**	Control Group	2.6 ± 1.53	-	0.001
	Combined Therapy	2.02 ± 1.33	0.001	-
	Tamsulosin	2.65 ± 1.54	0.25	0.62
	Mirabegron	2.33 ± 2.24	0.76	0.12
	Solifenacin	2.56 ± 1.56	0.73	0.76
	Desloratadine	2.71 ± 1.75	0.21	0.72
**Vas Pain**	Control Group	3.55 ± 2.55	-	0.38
	Combined Therapy	2.65 ± 1.69	0.38	-
	Tamsulosin	2.96 ± 1.55	0.16	0.88
	Mirabegron	2.78 ± 2.71	0.98	0.98
	Solifenacin	3.37 ± 1.99	0.35	0.34
	Desloratadine	3.12 ± 1.98	0.83	0.01
**General Health**	Control Group	5.64 ± 5.08	-	0.12
	Combined Therapy	5.76 ± 3.35	0.12	-
	Tamsulosin	4.69 ± 3.74	0.47	0.76
	Mirabegron	5.29 ± 4.88	0.69	0.45
	Solifenacin	5.22 ± 3.47	0.14	0.76
	Desloratadine	6.10 ± 3.20	0.13	0.49
**Work Performance**	Control Group	3.73 ± 3.90	-	0.88
	Combined Therapy	2.88 ± 3.42	0.88	-
	Tamsulosin	3.20 ± 4.27	0.61	0.58
	Mirabegron	4.00 ± 5.43	0.61	0.03
	Solifenacin	2.31 ± 4.71	0.88	0.22
	Desloratadine	3.39 ± 4.05	0.42	0.66
**Sexual life**	Control Group	0.72 ± 1.19	-	0.75
	Combined Therapy	0.67 ± 1.05	0.75	-
	Tamsulosin	0.80 ± 1.28	0.61	0.86
	Mirabegron	0.57 ± 0.87	0.13	0.37
	Solifenacin	0.59 ± 0.96	0.41	0.69
	Desloratadine	0.71 ± 1.20	0.61	0.25
**Additional Problems**	Control Group	2.39 ± 1.92	-	0.001
	Combined Therapy	2.12 ± 1.88	0.001	-
	Tamsulosin	2.22 ± 2.01	0.65	0.87
	Mirabegron	2.18 ± 2.33	0.34	0.30
	Solifenacin	2.24 ± 1.85	0.26	0.25
	Desloratadine	2.18 ± 2.43	0.49	0.09

## Data Availability

The data that support the findings of this study are available from the corresponding author upon reasonable request.
